# Infants’ and toddlers’ physical activity and sedentary time as measured by accelerometry: a systematic review and meta-analysis

**DOI:** 10.1186/s12966-020-0912-4

**Published:** 2020-02-07

**Authors:** Brianne A. Bruijns, Stephanie Truelove, Andrew M. Johnson, Jason Gilliland, Patricia Tucker

**Affiliations:** 1 Health and Rehabilitation Sciences Program, Faculty of Health Sciences, University of Western Ontario, London, Ontario Canada; 2 School of Health Studies, Faculty of Health Sciences, University of Western Ontario, London, Ontario Canada; 3 Department of Geography, Faculty of Social Sciences, University of Western Ontario, London, Ontario Canada; 4 School of Occupational Therapy, Faculty of Health Sciences, University of Western Ontario, 1201 Western Road, Elborn College, Room 2547, London, ON N6G 1H1 Canada

**Keywords:** Physical activity, Sedentary time, Infants, Toddlers, Young children, Accelerometer, Systematic review, Meta-analysis

## Abstract

**Background:**

Early experiences in physical activity (PA) are important to shape healthy movement behaviours long-term; as such, it is critical that PA is promoted from infancy, and that detrimental behaviours (e.g., prolonged sedentary time [ST]) are minimized. The purpose of this systematic review and meta-analysis was to examine infants’ and toddlers’ movement behaviours across daytime hours.

**Methods:**

Seven online databases were searched for terms related to infants (< 12 months), toddlers (12–35.9 months), PA, ST, and accelerometry. Two independent reviewers examined 4873 articles for peer-reviewed original research, published in English, that assessed infants’ (counts/min) and/or toddlers’ PA or ST (min/day) using accelerometry across daytime hours. Infants’ mean PA level (counts/min) was averaged across studies, and ranges were produced. Estimates of toddlers’ movement behaviours were aggregated meta-analytically to produce average daily rates, and accelerometer placement, cut-point validity, device type, and epoch length were tested as a moderating variables.

**Results:**

Twenty-four studies from 16 countries (published 2011–2019), representing 3699 participants, were included in the systematic review. Five studies reported on infants’ PA, which ranged from 78.2 to 2580.5 cpm. Across 20 studies, toddlers’ total PA, light PA, moderate-to vigorous-intensity PA, and ST ranged from 72.9 to 636.5, 48.5 to 582.4, 6.5 to 89.9, and 172.7 to 545.0 min/day, respectively. After taking into account accelerometer placement, cut-point validity, device type, and epoch length, we found that toddlers engaged in 246.19 min/day (*SE* = 28.50; 95% CI: 190.34, 302.04) of total PA, 194.10 min/day (*SE* = 28.76; 95% CI: 137.73, 250.47) of light PA, and 60.16 min/day (*SE* = 5.88; 95% CI: 48.64, 71.69) of moderate-to vigorous-intensity PA. Toddlers engaged in 337.04 min/day (*SE* = 32.67; 95% CI: 273.01, 401.07) of ST.

**Conclusions:**

With limited studies conducted in infants (*n* = 5), PA estimates are inconclusive and largely heterogeneous. Overall, toddlers tend to exceed the total PA recommendation of 180 min/day; however, very little of this time is spent at higher movement intensities. Even with high PA rates, toddlers still engage in substantial ST. More consistent and valid measurement protocols are needed to improve comparability across studies.

## Background

Establishing healthy movement behaviours in early childhood is necessary to support the development and maintenance of long-term health [1, 2]. The benefits of physical activity in early childhood are abundant and include improved cardiometabolic biomarkers [3], mental health [4], and both cognitive [5] and social [3, 6] development [7]. Specifically, in infants (i.e., < 12 months), physical activity is associated with improved adiposity measures and motor skill development [3]. In toddlers (i.e., 12–35.9 months), this behaviour is associated with improved bone and skeletal health. On the contrary, high levels of sedentary behaviours in the early years negatively impact children’s health [8, 9]. In particular, screen-viewing among young children (< 4 years) is related to decreased psychosocial health and cognitive development, irregular sleep patterns, and increased adiposity [9]. Considering activity habits developed in early childhood tend to carry into later childhood and adolescence [1, 2], promoting healthy movement behaviours from the beginning of life is highly important.

Emerging evidence indicates that the interaction of movement behaviours (including sleep, sedentary behaviour, light physical activity [LPA], and moderate-to vigorous-intensity physical activity [MVPA]) across each 24-h day has important health implications [10]. As such, age-appropriate recommendations have been developed to help different cohorts achieve optimal movement profiles for their health. According to *The Canadian 24-Hour Movement Guidelines for the Early Years* [11], infants should engage in floor-based play throughout the day with a minimum of 30 min/day of tummy time, while toddlers should engage in 180 min/day of total physical activity (TPA), including at least some energetic play (i.e., MVPA). Additionally, screen time is not recommended for those under 2 years and should be limited to less than 1 h/day for those over 2 years [11]. Furthermore, children should not be sedentary or restrained for more than 1 h at a time [11]. Other countries (e.g., Australia [12], New Zealand [13]), as well as the World Health Organization (WHO) [14], have adopted similar recommendations for these age groups, endorsing an integrated approach. As such, these guidelines can act as important benchmarks to compare young children’s daytime movement behaviours globally and should be taken into consideration when assessing whether infants and toddlers are engaging in appropriate daily physical activity and sedentary time to benefit their health.

A review by Cardon and colleagues (2011) explored infants’ and toddlers’ physical activity and sedentary behaviours and yielded only six papers, none of which used accelerometry as a measurement tool [15]. With only two studies assessing this population’s physical activity behaviours, no conclusions could be drawn; however, observational and survey-based studies highlighted the high prevalence of screen-viewing among children under 2 years, warranting further investigation. Since this review, the use of accelerometry to assess infants’ and toddlers’ movement behaviours has become more common. A scoping review by Prioreschi et al. [16] in 2016 summarized physical activity in children under 2 years. Given the heterogeneity in measurement tools used (e.g., accelerometer, motion sensor, metabolic chamber, direct observation, etc.), synthesis of results was not possible [16]. Further, only six studies reported actual physical activity levels of children under 2 years, three by way of accelerometry; as such, no concrete conclusions could be drawn [16]. The authors of these two reviews stressed the importance of using accelerometry, the gold standard in the objective measurement of infants’ and toddlers’ physical activity [17], to allow for comparisons across studies.[15, 16] Additionally, considering young children’s activity patterns are often sporadic in nature, typically involving short bursts of movement [18], using short epoch lengths is essential in order to capture this population’s true activity behaviours [15, 19, 20].

Following such recommendations, a recent systematic review and meta-analysis by Pereira and colleagues [21] assessed the prevalence of accelerometry-measured sedentary behaviours among young children 2 to 6 years of age. Across 47 studies, children spent approximately 55% of their time sedentary. While this review presented important findings regarding levels of sedentary time between boys and girls, weekdays and weekend days, and childcare hours and out-of-care hours, toddlers’ and preschoolers’ sedentary time were summarized together. Additionally, a minimum accelerometer wear time criterion was not applied, reducing the likelihood that the sedentary behaviour rates produced accurately reflect daily habitual levels. Although a systematic review by Downing and colleagues [22] summarized the sedentary behaviours of children < 2 years, no included studies used objective assessments, resulting in substantial variation in daily estimates (which ranged from 36.6 to 330.9 min/day). Evidently, a summary of accelerometry-measured sedentary time of young children < 3 years is needed.

With the rapid influx of physical activity and sedentary behaviour research transpiring among this young cohort, particularly with accelerometry, a synthesis of this literature was needed. Conducting such analyses would allow for direct comparison to recommendations within internationally recognized movement guidelines for these developmental age groups and would provide valuable findings to inform future interventions to support young children’s development of healthy activity habits. As such, the aim of this systematic review and meta-analysis was to summarize infants’ and toddlers’ daily physical activity and sedentary time as measured by accelerometry.

## Methods

This review was registered with the International Prospective Register of Systematic Reviews (registration no. CRD42018114477) and adheres to the Preferred Reporting Items for Systematic Reviews and Meta-Analyses (PRIMSA) statement for systematic reviews.

### Search strategy

Seven online databases (PubMed, Physical Education Index, Sport Discus, PsychINFO, CINAHL, SCOPUS, and EMBASE) were systematically searched using terms related to “infant”, “toddler”, “physical activity”, “sedentary behaviour”, and “accelerometry”. See Additional file 1: Table S1 for a sample search strategy. No date restrictions were used; however, due to the requirement of accelerometry, a natural restriction was generated based on the first appearance of this device in research. The initial database searches were completed on October 29, 2018, with an updated search undertaken on October 9, 2019. All retrieved papers were exported into a unique folder in Mendeley© (v1.19) referencing software and duplicates were removed.

### Eligibility criteria

To be eligible for inclusion in this systematic review, studies needed to meet the following criteria: be original research; published in English in a peer-reviewed journal; focus on typically developing (i.e., free from chronic disease and/or developmental issues) infants (< 12 months) and/or toddlers (12–35.9 months); and, measure physical activity and/or sedentary time via accelerometry (separately for infants and toddlers) across daytime hours (i.e., > 7 h of wear time, validated in the literature to reflect habitual activity levels of toddlers) [23]. Additionally, infant studies needed to present accelerometry data in counts/min (or provide sufficient information for calculations to be made), as valid cut-points to classify movement intensities for this population do not exist. To allow for comparison to movement guidelines for the toddler age group, physical activity and/or sedentary time needed to be presented as min/day (or provide sufficient information for calculations to be made). To be considered for inclusion in the meta-analysis, papers needed to meet the above criteria, provide the standard deviation for any intensity-specific activity data (or sufficient information to calculate these), and state the sample size.

Following pre-screening of titles (BAB), two reviewers (BAB, ST) independently assessed the titles and abstracts of potentially relevant articles. All articles passing this stage of eligibility by either reviewer were included in the full-text review process. The same two reviewers read each paper in full to determine appropriateness of inclusion, and reasons for exclusion were noted. In cases of uncertainty, a third reviewer (PT) was brought in for consultation, and a final list of articles was generated. In order to confirm all relevant and up to date literature was captured, the reference lists of all included articles, as well as the ahead of print/in-press sections of four journals (i.e., *Pediatric Exercise Science*, *Journal of Physical Activity and Health*, *International Journal of Behavioural Nutrition and Physical Activity*, and *BMC Public Health*) were examined. In cases where more than one article presented baseline data on the same sample of children (Melbourne InFANT Program [23], GET UP! [24], POI [25], IDEFICS [26], Generation R [27], Early STOPP [28], and PREPS [29]), the article with the largest sample size and/or most accurately reflected full day movement behaviours was included.

### Data extraction

The following information was extracted from each included article: 1. study characteristics (i.e., authors, publication year, country, study design, sample characteristics); 2. accelerometry details (i.e., accelerometer type, placement, epoch length used, average wear time, monitoring time, cut-points applied); and 3. outcome variable (i.e., physical activity [counts/min or TPA, LPA, MVPA] and/or sedentary time). In order to accurately reflect habitual physical activity and sedentary time, only baseline or control group data of intervention studies were extracted. For papers comparing typically developing young children with atypically developing children, only data from the former group was included. If any data for extraction were missing, authors were contacted.

### Quality assessment and risk of Bias

Two independent reviewers (BAB, ST) assessed the quality and risk of bias of included studies using the Downs and Black checklist [30]. A third reviewer (PT) was consulted in cases of disagreement. Considering only cross-sectional data was pulled for the purposes of this study (e.g., only baseline/control group data from intervention studies were included), a modified version of the checklist was used (i.e., questions 1–3, 6, 7, 10–12, 18, and 20), consistent with previous research [31, 32]. Articles were scored as either low (i.e., 0–3), medium (i.e., 4–6), or high (i.e., 7–10) quality.

### Data synthesis and analysis

Infant and toddler activity levels were synthesized separately due to differences in data presentation (i.e., counts/min vs. min/day) and typical accelerometer placement (i.e., wrist/ankle vs. waist), and studies in each age category were grouped by country to facilitate intra- and inter-country comparisons. If not already provided, infant physical activity data were converted to counts/min. Given movement intensity cut-points have not yet been developed or validated in infants, sedentary time could not be explored in this cohort. Toddler physical activity (TPA, LPA, and MVPA) and sedentary time data were converted to min/day, using simple calculations (e.g., adding LPA and MVPA to produce TPA). Weighted means were produced for studies not presenting total sample data for the target population (e.g., data for boys and girls were presented separately).

Accelerometer results for toddlers were combined using meta-analytic techniques, on four variables: TPA, LPA, MVPA, and sedentary time. All intensities were measured in minutes. One study [33] was excluded from analysis, as it presented interquartile range (IQR) in place of a standard deviation (SD); while it is possible to estimate SD from the IQR (e.g., IQR/1.35), this is generally only possible when the data are normally distributed. Given IQR is typically only presented in lieu of SD when the data are grossly non-normal, no SD was recorded in the data set for this study.

Four moderators were identified for inclusion in the analysis: device (ActiGraph versus Actical), epoch length (15 s or less, or more than 15 s), use of a set of cut-points validated in the toddler age group (yes or no), and accelerometer placement (ankle, waist, and wrist). In a separate meta-analysis of each of the four outcome variables, we fit a mixed effects model that tested the overall effect of all four moderators using Cochran’s Q. The reference conditions chosen within this moderator analysis (for the purposes of describing overall effects) were: 1. studies that used ActiGraph accelerometers; 2. studies with an epoch length of 15 s or less; 3. studies that employed a set of cut-points validated in toddlers for their analysis; and, 4. studies that placed the accelerometer on the waist of the participant. Heterogeneity was estimated using a restricted maximum-likelihood estimator, and the statistical significance of residual heterogeneity was carried out using Cochran’s Q-test. Analyses were conducted in R version 3.6.1 [34], using meta-analytic functions from the metafor package [35].

## Results

### Database searches

**Fig. 1:**
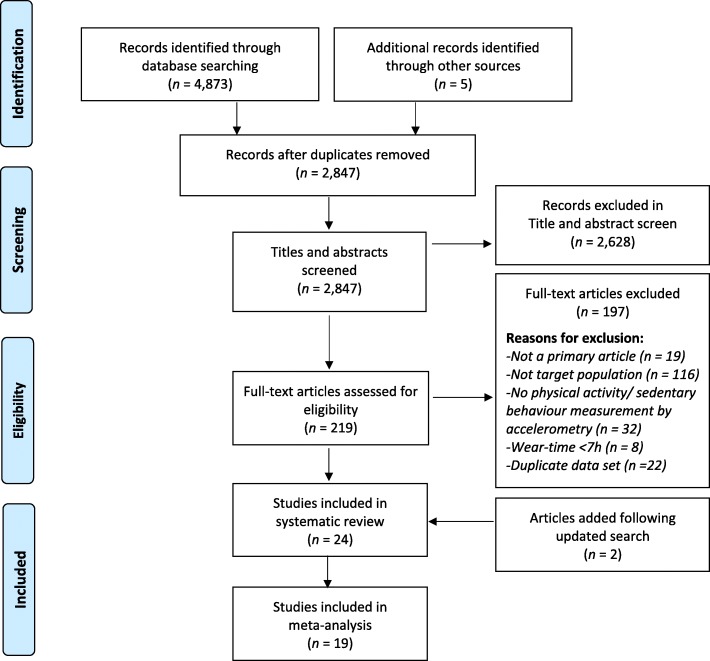
Flow diagram representing the identification, screening, eligibility, and inclusion of studies in this review and meta-analysis

The systematic database search identified 4873 records. After removing duplicates, 2845 articles underwent title pre-screening to remove studies that did not focus on typically developing young children. Following title and abstract screening of 236 articles, 215 papers were reviewed for eligibility in full and 22 met the inclusion criteria. Following an update of the search (for articles published after October 29, 2018), 2 additional articles met the inclusion criteria. Of the 24 included studies, 19 were included in the meta-analysis. See Fig. 1 for a flow diagram of the identification and screening process, as well as the number of articles excluded per exclusion criterion.

### Study characteristics and quality of included articles

Studies were conducted in 16 different countries, including 8 in the United States [36–43], 4 in Canada [29, 44–46] and Australia [23, 24, 47, 48], 2 in China [28, 49] and Sweden [26, 28], and 1 in Belgium [26], Estonia [26], Germany [26], Hungary [26], Italy [26], Malawi [50], the Netherlands [27], New Zealand [25], Spain [26], Switzerland [33], and Taiwan [51] (note that one study was conducted in both China and Sweden [28] and one study was conducted in Belgium, Estonia, Germany, Hungary, Italy, Spain, and Sweden [26]). The mean sample size of included studies was 142 children (range = 7 to 568), and all studies were published in 2011 or later (88% since 2015). All studies were of high quality (i.e., a score of 7–10 on the modified checklist); however, this was not established a priori. See Tables 1 and 2 for study characteristics and quality ratings for infant and toddler studies, respectively. The full quality rating scores can be found in Additional file 2: Table S2.

**Table 1:** Summary Table for Included Studies - Infants (<12mo; *n* = 5)

Authors (Year)	Study Design	Sample Size, Mean Age (mo; *SD*), Range	Average Wear-Time min/day (*SD*), Non-Wear Criteria	Monitoring Time	Accelerometer Type & Epoch Length	Accelerometer Placement	Physical Activity counts/min (*SD*)	Study Quality
*Canada (n = 1)*								
Borkhoff et al. (2015) [45]	Cross-sectional	*N* = 20	552 (54), ≥60 min of consecutive zeros	12 h, 7 days	Actical, 2-s	Waist	78.2 (30.8)	10
		Age = 8.2 (1.8), 4–10						
*China (n = 1)*								
Jia et al. (2018) [49]	Cross-sectional	*N* = 143	NR, NR	14 h, 7 days	ActiGraph GT3X, 60-s	Wrist	2580.5 (475.7)	10
		Age = 9 (0.8), 9						
*Taiwan (n = 1)*								
Wang et al. (2019) [51]	Cross-sectional	*N* = 183	NR, ‘Device removal’ time replaced with mean activity counts	24 h, 7 days	Actiwatch 2, 30-s	Ankle	432.3 (124.8) ^a^	10
		Age = 6.6 (0.4), 6						
*United States (n = 2)*								
Tsai et al. (2011) [40]	Cross-sectional	*N* = 22	NR, ‘Device removal’ replaced with the adjacent activity counts, external motion time was removed from analysis	16 h, 7 days	Actiwatch, 30-s	Ankle	144.0 (66.0) ^a^	9
		Age = 1.6 (0.6), 0.5–2.5						
Pitchford et al. (2017) [39]	Cross-sectional	N = 23	556.2 (157.2), ≥2 min of consecutive zeros matching accelerometer logged ‘device removal’ within a 30-min window	Daytime, 7 days	ActiGraph GT3X, 15-s	Ankle and Wrist	1758.6 (609.6) ^a b^	9
		Age = 6.3 (3.6), 1–12						

*NR* not reported, *mo* months; ^a^Calculation made to convert to counts/min; ^b^Mean of ankle and wrist counts produced

**Table 2:** Summary Table for Included Studies - Toddlers (12–35.9mo; n = 20)

Authors (Year)	Study Design	Sample Size, Mean Age (mo; *SD*), Range	Average Wear-Time min/day (*SD*), Non-Wear Criteria	Monitoring Time	Accelerometer Type, Placement, Epoch Length	Cut-Points	Physical Activity min/day (*SD*)	Sedentary Time min/day (*SD*)	Study Quality
*Australia (n = 4)*									
Hnatiuk et al. (2012) [23]	Cluster RCT	*N* = 295	586.4 (65.1), ≥20 min of consecutive zeros	Waking hours, 7 days	ActiGraph GT1M, Waist, 15-s	Trost et al.	TPA^Φ^ = 232.4 (34.1)	NR	10
		Age = 19.1 (2.3), NR							
							LPA = 184.5 (30.7)		
							MVPA = 47.9 (16.2)		
Hnatiuk et al. (2017) [47]	Cross-sectional	*N* = 136	658.6 (94.8), ≥20 min of consecutive zeros	Waking hours, 7 days	ActiGraph GT3X, Waist, 15-s	Trost et al.	TPA = 303.8 (53.9)	NR	8
		Age = 32.4 (10.2), 12–36							
							LPA = 228.4 (34.8)		
							MVPA = 75.4 (27.3)		
Oftedal et al. (2015) [48]	Cohort	N = 20	571 (72), Consecutive zeros matching accelerometer logged ‘device removal’	Waking hours, 3 days	ActiGraph GT3X, Waist, 5-s	Oftedal et al.	NR	279.8 (28.6) ^Φ^	9
		Age = 26.4 (6.0), 18–36							
Santos et al. (2017) [24]	RCT	*N* = 202	1297.4 (93), Accelerometer logged ‘device removal’ (only full 24 h days included in analyses)	24 h, 7 days	ActiGraph GT3X+, Waist, 15-s	Trost et al.	TPA = 295.0 (59.0)	262.8 (60.2)	10
		Age = 19.7 (4.1), 12–28							
							LPA = 237.7 (44.2)		
							MVPA = 57.3 (21.4)		
*Canada (n = 4)*									
Bisson et al. (2018) [44]	Cohort	*N* = 255	527.1 (174.0), ≥20 min of consecutive zeros	Waking hours, 7 days	ActiGraph GT3X, Waist, 15-s	Trost et al.	TPA = 223.3 (80.4)	NR	10
		Age = 25.2 (2.4), NR							
Borkhoff et al. (2015) [45]	Cross-sectional	*N* = 27	582 (48), ≥60 min of consecutive zeros	12 h, 7 days	Actical, Waist, 2-s	Adolph et al.	TPA = 193.8 (63.7)	385.9 (52.4)	10
		Age = 19.1 (5.3), 12–30							
						Wong et al.	LPA = 185.1 (57)		
							MVPA = 8.7 (12)		
Lee et al. (2017) [29]	Cross-sectional	*N* = 151	618 (84), ≥20 min of consecutive zeros	Waking hours, 7 days	ActiGraph wGT3X-BT, Waist, 15-s	Trost et al.	TPA = 298.9 (40.9)	316.7 (40.6)	9
		Age = 19 (1.9), 12–23				Pate et al.			
							LPA = 240.2 (29.3)		
							MVPA = 58.7 (18.7)		
Vanderloo et al. (2015) [46]	Cross-sectional	N = 40	606.8 (38.8), ≥60 min of consecutive zeros	Waking hours, 7 days	Actical, Waist, 15-s	Trost et al.	TPA^Φ^ = 107.2 (33.3)	499.43 (33.3) ^Φ^	10
		Age = 25.7 (5.9), 18–29							
							LPA^Φ^ = 99.0 (29.3)		
							MVPA^Φ^ = 8.29 (7.3)		
*China (n = 1)*									
Johansson et al. (2016) [28]	Cross-sectional	*N* = 79	NR - min 15 h to be valid, NR	15 h, 7 days	ActiGraph GT3X, Wrist, 5-s	Johansson et al.	TPA^Φ^ = 235 (83.2)	545.0 (99.0)	10
		Age = 24.5 (1.6), NR							
							LPA = 195.0 (79.0)		
							MVPA = 40.0 (26.0)		
*Malawi (n = 1)*									
Pulakka et al. (2017) [50]	RCT	*N* = 380	810, ≥20 min of consecutive zeros	15 h, 5 days	ActiGraph GT3X+, Waist, 15-s	Trost et al.	MVPA^Φ^ = 89.9 (28.4)	432.5 (66.4) ^Φ^	10
		Age = 18.1 (0.6), NR							
*Netherlands (n = 1)*									
Wijtzes et al. (2013) [27]	Cohort	*N* = 347	488, ≥10 min of consecutive zeros	24 h, 1 weekday, 1 weekend day	ActiGraph Am-7164, Waist, 15-s	Sirard et al.	TPA^Φ^ = 72.9 (28.3)	415.1 (28.3) ^Φ^	9
		Age = 25.1 (1.1), NR							
							LPA^Φ^ = 48.5 (16.1)		
							MVPA^Φ^ = 24.4 (14.6)		
*New Zealand (n = 1)*									
Taylor et al. (2018) [25]	RCT	*N* = 568	NR - min 20 h to be valid, ≥20 min of consecutive zeros	24 h, 7 days	Actical, Waist, 15-s	Pfeiffer et al.	TPA^Φ^ = 228.0 (61.4)	519.8 (62.0) ^δ^	9
		Age^δ^ = 16.7 (0.3), 12–35.9							
						Adolph et al.	LPA^δ^ = 221.5 (61.0)		
						Trost et al.	MVPA^δ^ = 6.5 (7.1)		
*Sweden (n = 1)*									
Johansson et al. (2016) [28]	Cross-sectional	*N* = 146	NR - min 15 h to be valid, NR	15 h, 7 days	ActiGraph GT3X, Wrist, 5-s	Johansson et al.	TPA^Φ^ = 334.0 (40.2)	445.0 (68.0)	10
		Age = 24.4 (1.2), NR							
							LPA = 261.0 (49.0)		
							MVPA = 73.0 (29.0)		
*Switzerland (n = 1)*									
Herzig et al. (2017) [33]	Cross-sectional	*N* = 19	NR - min 10 h to be valid, ≥20 min of consecutive zeros	14 h, 8 days	ActiGraph wGT3X, Waist, 30-s	Butte et al.	MVPA = 54.8 (NR)	NR	8
		Age = 33.6 (NR), 31.2–34.8							
*United States (n = 6)*									
Armstrong et al. (2018) [36]	Cross-sectional	*N* = 195	NR - min 24 h to be valid, NR	24 h, 7 days	Actical, Ankle, 60-s	Hager et al.	NR	172.7 (39.6)	10
		Age = 20.3 (5.6), 12–32							
Dlugonski et al. (2017) [37]	Cross-sectional	N = 7	672.4 (74), Accelerometer logged ‘device removal’	Waking hours, 7 days	ActiGraph GT3X, Waist, 60-s	Trost et al.	TPA^Φ^ = 294.2 (64.9)	NR	9
		Age = NR, 12.0–35.9							
							LPA = 257.4 (61.3)		
							MVPA = 36.8 (21.3)		
Hager et al. (2016) [38]	Cross-sectional	*N* = 191	NR - min 24 h to be valid, NR	24 h, 7 days	Actical, Ankle, 60-s	Hager et al.	TPA^Φ^ = 636.5 (96.4)	NR	9
		Age = 20.1 (NR), 12–32							
							LPA = 582.4 (87.5)		
							MVPA = 54.1 (40.4)		
Hauck & Kim (2019) [41]	Cross-sectional	*N* = 35	503.8 (146.4)^δ^, ≥20 min of consecutive zeros	At least 3 h, at least 3 days (avg. 6.0 monitoring days ^δ^)	ActiGraph GT3X	Trost et al.	NR	348.0 (95.3) ^δ^	10
		Age = NR, 18-24			Waist, 15-s				
Kwon et al. (2019) [42]	Cross-sectional	N = 19	499.0^a^ (NR), Accelerometer logged ‘device removal’ and ≥ 20 min of consecutive zeros	Waking hours, at least 4 days (1 weekend day)	ActiGraph GT3X	Trost et al.	TPA^Φ^ = 208.0 (30.0)	NR	10
		Age = NR, 12-35			Waist, 15-s				
							LPA = 161.0 (26.0)		
							MVPA = 47.0 (15.0)		
McCullough et al. (2018) [43]	Cross-sectional	*N* = 65	606 (78), ≥20 min of consecutive zeros	Waking hours, 7 days	ActiGraph, Waist, 15-s	Trost et al.	TPA = 293.7 (60.1)	312.6 (61.3)	10
		Age = 29 (4), 24–35							
							LPA^Φ^ = 232.4 (53.4)		
							MVPA = 61.3 (27.6)		
*Other - Europe (Multi-Country: Italy, Estonia, Belgium, Sweden, Germany, Hungary, Spain; n = 1)*									
Konstabel et al. (2014) [26]	Cohort	*N* = 131	740 (100), ≥20 min of consecutive zeros	Waking hours, at least 3 days (1 weekend day)	ActiGraph (GT1M, ActiTrainer), Waist, 15-s and 60-s	Evenson et al.	TPA^δ^ = 435.8 (60.3)	239.2 (68.8) ^δ^	9
		Age = NR, 24–35.9							
							LPA^δ^ = 411. 8 (58.9)		
							MVPA^δ^ = 24.0 (13.1)		

*RCT* randomized control trial, *mo* months, *TPA* total physical activity, *LPA* light physical activity, *MVPA* moderate-to vigorous-intensity physical activity, *NR* not reported; ^Φ^Calculation made to produce value; ^δ^Weighted mean produced; ^a^Median wear time

Five studies reported on the physical activity levels of infants and 17 reported on physical activity levels of toddlers. Thirteen studies reported on the daily sedentary time of toddlers. Three brands of accelerometers were used to objectively measure children’s movement behaviours: ActiGraph™ (*n* = 17); Actical™ (*n* = 5); and, Actiwatch™ (*n* = 2). The majority (90%) of included studies used waist placement of the accelerometer. Average accelerometer wear time ranged from 8.1 to 24 h per day, with a range of 2 to 8 monitoring days. Epoch lengths varied across studies, with two-thirds using an epoch of 15 s or less (as recommended for activity measurement in infants and toddlers [18, 19]). Cut-points were not applied in the infant studies, as they have not yet been validated for this age group. Cut-points used in the toddler studies varied, with Trost et al.’s cut-points [52] most frequently applied (*n* = 11). Five studies [25–27, 33, 45] applied cut-points not validated for the toddler age group. See Table 3 for a summary of accelerometry characteristics.

**Table 3:** Summary of Accelerometer Characteristics of Included Studies (*n* = 24)

Accelerometer Characteristic	Number of Studies
Model	
ActiGraph	17
Actical	5
Actiwatch	2
Epoch Length	
5-s or less	3
15-s	13
30-s	3
60-s	5
Average Wear-Time	
7–9.9 h	8
10–15 h	7
> 15 h	1
Not reported	8
Number of Monitoring Days	
3 or less	3
4–6	3
7 or more	18
Cut-Points Applied^a^	
Trost et al.	11
Oftedal et al.	1
Adolph et al.	1
Johansson et al.	1
Sirard et al.	1
Butte et al.	1
Hager et al.	2
Evenson et al.	1
Multiple	1

^a^Cut-points were applied in toddler studies only (*n* = 20)

### Infants’ physical activity

Due to significant heterogeneity in infants’ physical activity counts/min (*p* = <.0001), meta-analysis of these data was not appropriate. Infants’ mean physical activity level was 1494.4 cpm, and ranged from 78.2 cpm to 2580.5 cpm. Three studies were conducted in North America (Canada [*n* = 1], and United States [*n* = 2]), and two studies were conducted in Asia (China [*n* = 1], and Taiwan [*n* = 1]). See Table 1 for a summary of infant physical activity data.

### Toddlers’ physical activity and sedentary time

Fifteen studies reported on toddlers’ *TPA*, with estimates ranging from 72.9 to 636.5 min/day. Fourteen studies reported on toddlers’ *LPA,* which ranged from 48.5 to 582.4 min/day. Sixteen studies reported on toddlers’ *MVPA*, and estimates ranged from 6.5 to 89.9 min/day. Across 13 studies, toddlers spent 172.7 to 545.0 min/day engaged in sedentary behaviour. See Table 2 for estimates of TPA, LPA, MVPA and sedentary time.

**Fig. 2:**
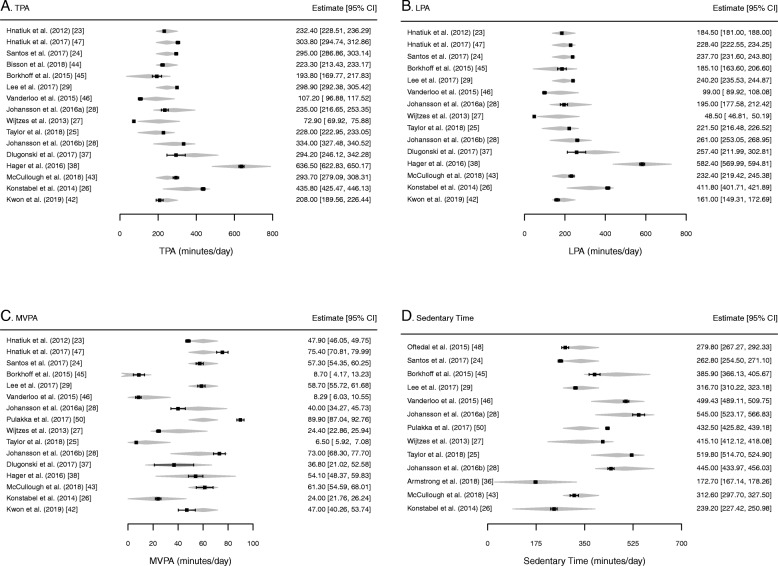
Forest plot of meta-analyses moderated by accelerometer placement, validity of cut-point, type of accelerometer, and epoch length. The polygon presented along with the effect size estimates for each study in the meta-analysis, depicts the fitted estimate. The width of this polygon represents the confidence interval surrounding this fitted estimate. *TPA* total physical activity, *LPA* light physical activity, MVPA moderate-to vigorous-intensity physical activity

Cochran’s Q indicated that there was a statistically significant effect associated with the four moderators included within the model, for TPA [Q(5) = 30.90, *p* < .001], LPA [Q(5) = 31.29, p < .001], and MVPA [Q(5) = 26.92, p < .001]. After taking into account accelerometer placement, cut-point validity, device type, and epoch length, we found that toddlers engaged in 246.20 min/day (*SE* = 28.50; 95% CI: 190.34, 302.04) of total PA, 194.10 min/day (*SE* = 28.76; 95% CI: 137.73, 250.47) of light PA, and 60.16 min/day (*SE* = 5.88; 95% CI: 48.64, 71.69) of moderate-to vigorous-intensity PA. Toddlers engaged in 337.04 min/day (*SE* = 32.67; 95% CI: 273.01, 401.07) of sedentary time. This information is summarized in Table 4 and Fig. 2.

**Table 4:** Summary of Meta-Analytic Results for Toddlers’ Physical Activity and Sedentary Time

	*n*	# studies	Estimate (min/day)	*SE*	95% CI
Sedentary Time	2351	13	337.04	32.67	273.01 to 401.07
LPA	2404	14	194.10	28.76	137.73 to 250.47
MVPA	2784	15	60.16	5.88	48.64 to 71.69
TPA	2659	15	246.19	28.50	190.34 to 302.04

*SE* standard error, *CI* confidence interval, *LPA* light physical activity, *MVPA* moderate-to vigorous-intensity physical activity, *TPA* total physical activity

## Discussion

This was the first systematic review and meta-analysis to explore accelerometry-measured physical activity of infants and toddlers, as well as sedentary time of toddlers, across daytime hours. While infants’ daily physical activity remains unclear (due to the complexity of objectively measuring and analyzing infant locomotor activity), toddlers appear to be achieving their recommended 180 min/day of TPA. Given the rapid growth in early years physical activity and sedentary behaviour research, coupled with developments in accelerometry measurement protocols for young children, this synthesis of the literature provides a necessary snapshot of this population’s daytime movement behaviours. With increased attention being placed on 24-h movement behaviours globally, this review makes an important contribution to the current literature by providing movement behaviour estimates (of toddlers’ physical activity and sedentary time) that can be compared to recommendations within the recently released *24-Hour Movement Guidelines* [11].

### Infants’ movement Behaviours

With the lack of existing validated cut-points for infants, activity data were summarized in counts/min to allow for direct comparison across studies. Even in this raw form, heterogeneity in physical activity levels remained evident. The lower accelerometer counts produced by Borkhoff et al. [45], Tsai et al. [40], and Wang et al. [51] may have been influenced by their use of the Actical™ and Actiwatch™ devices, which typically produce lower activity counts than the ActiGraph™ [53, 54] (as used by Jia et al. [49] and Pitchford et al. [39]). Further, Borkhoff and colleagues’ [45] low activity counts were likely affected by their use of waist placement of the accelerometer, which cannot capture limb movement by infants who are unable to walk. As noted by Pitchford and colleagues [39], accelerometer placement on the wrist and/or ankle is critical to capture this limb movement; however, activity recording at these two locations significantly differs. In this reliability study [39], activity counts were higher when recorded at the wrist compared to the ankle; in the present review, the study by Jia et al. [49] used only wrist accelerometer placement and produced the highest counts/min rate, whereas Tsai et al. [40] used only ankle accelerometer placement and produced the second lowest counts/min rate. Ricardo and colleagues [55] recently developed a protocol for wrist and ankle accelerometer use in infants, and found that 2 and 3 days of measurement were needed at these locations to capture physical activity levels, respectively. However, it should be noted that Tsai and colleagues [40] used a sample population of 2- to 10-week-old infants, while the remainder of the infant studies used samples ranging from 1- to 12-month-old infants. As such, age may have been a factor influencing this outcome, as daily activity levels increase across the first year of life as infant sleep-wake cycles regulate [56] and motor development progresses [57]. Additionally, infant studies adopted a variety of epoch lengths (2 s [45], 15 s [39], 30s [40, 51], and 60s [49]), which is a known contributing factor to variability in activity measurement in older cohorts [19, 20]. Future research is needed to manage the difficulties encountered when using accelerometry in this young population.

### Toddlers’ movement behaviours

Toddlers’ physical activity levels were more easily interpreted, with available cut-points allowing for meaningful translation of activity data. In general, toddlers reported to be exceeding the TPA recommendation of 180 min/day. While this is encouraging, the majority of study populations (~ 75%) reported MVPA estimates below the recommended 60 min/day of MVPA children should engage in by the age of 3 [11]. Considering research has shown that young children’s activity levels begin declining as early as 3 years of age [58], there is room for improvement for toddlers to get set on the right trajectory. While LPA does produce many important health benefits for this young cohort, such as improved cardiometabolic health [7], engaging in MVPA presents health benefits over and above what LPA can provide. Such benefits include increased motor competence [59], improved bone health [60], and enhanced cognitive development [5]. As such, evidence suggests that in order to produce more favourable movement profiles of young children, replacing sedentary time with LPA, and LPA with MVPA, would provide substantial health benefits [61]. Specifically, in toddlers, who may not be able to maintain high intensity activity for long durations [62, 63], MVPA can also be introduced intermittently to break up bouts of sedentary behaviour; this may help mitigate the detrimental effects that long, uninterrupted bouts of sedentary time can have on children [64].

Despite adequate levels of TPA being reported among toddlers, sedentary time remained high. During waking hours, this was the most prevalent movement behaviour; as such, particular attention should be placed on whether sedentary bouts and activities (unable to be assessed in this review) are in line with recommendations for toddlers (i.e., < 1 h bouts of sedentary time, no screen time < 2 years, and engaging in developmentally appropriate sedentary pursuits [e.g., reading, drawing, etc.] [11]). Two of the included studies explored toddlers’ sedentary bouts in comparison to recommendations. Santos et al. [24] reported that in a sample of 202 Australian toddlers, no children engaged sedentary bouts lasting longer than 1 h, whereas Lee et al. [29] reported that only 34% of their sample of 151 Canadian toddlers met this requirement. Important to note, however, is that in the former study [24], sedentary bouts were measured by accelerometry, while the latter study [29] relied on parent-report data. Additionally, 4 studies in this review reported on toddlers’ screen-viewing behaviours [24, 27, 29, 46]. For toddlers less than 2 years of age, no screen-viewing is recommended; however, only 11.4% of Australian toddlers [24], and 15.2% [29] and 20.5% [46] of Canadian toddlers, met this screen time recommendation. Further, while less than 1 h of screen-viewing is recommended for toddlers over 2 years, approximately 14.2% of Dutch toddlers (*n* = 334) [27] and 68.0% of Canadian toddlers (*n* = 40) [46] met this guideline. While studies included in this review did not report on other sedentary behaviours that may be beneficial to toddlers’ achievement of developmental milestones (e.g., storytelling, circle time, reading) [65], the low prevalence of toddlers meeting their respective screen time recommendations is worrisome, as screen-viewing is associated with additional health concerns independent from sedentary time (e.g., irritable sleep, decreased cognitive and psychosocial health) [9], and this behaviour has been shown to track into later childhood and adolescence [66]. In light of the detrimental effects of screen-viewing in the early years [9], as well as the combined effect of movement behaviours on health markers [67], efforts should be made to reduce the amount of screen-based sedentary time that toddlers engage in daily.

### Methodological considerations

As is often the case with accelerometry-measured physical activity and sedentary time, methodological characteristics of the individual studies, such as cut-points applied, device type and placement, epoch length, and accelerometer wear time, can profoundly affect movement behaviour estimates in young children [18, 53]. Although cut-points in toddlers have only recently been validated (i.e., using the ActiGraph™ device) [52], the use of cut-points not validated in toddlers to reduce accelerometry data in this population remains problematic. In the present study, this was typically in cases where a wider age range of young children participated in the study (e.g., 0.3–5.8 years [45], 1–5 years [25], 2–6 years [33], and 2–10 years [26]). Choice of cut-points is critical, as the ActiGraph™ counts/15 s cut-point for MVPA in toddlers (> 418 counts/15 s [52]) has a lower threshold than that of Evenson et al. [68] and Sirard et al. [69] at > 574 and > 891 counts/15 s, respectively, which would result in more MVPA reported. In early years research where movement patterns change and develop substantially [18], a universal set of cut-points within a validated measurement protocol in toddlers would aid in producing more accurate movement behaviour estimates and ease comparability across studies.

In addition to cut-points applied, the accelerometer device type and placement also influences toddlers’ movement behaviour estimates. While validation work regarding device placement is limited in the toddler age group, waist placement provides the most precise estimates of young children’s full body movement (as compared to other wear locations [ankle, wrist, back]) and is most commonly used among this cohort [18]. Of note, Hager et al. (2016) and Armstrong et al. (2018) used ankle accelerometer placement, and reported physical activity estimates considerably higher, and sedentary time estimates considerably lower, than the remaining studies. Further, device type has also been noted to influence movement behaviour estimates; Vanderloo and colleagues [53] found that when comparing the Actical™ and ActiGraph™ in a sample of preschoolers (*n* = 23); the ActiGraph™ reported 6.6 more min/hour of MVPA than the Actical™. This hourly discrepancy would result in substantial variation in MVPA across an entire day; as evidenced by the present review, the 3 lowest MVPA rates reported (i.e., 6.5 [25], 8.3 [46], and 8.5 [45] min/day) were all from studies using the Actical™ device. While these rates may have also been a function of the epoch length used and intensity cut-points applied, interpreting physical activity data from studies using different devices and wear locations remains a challenge that needs to be addressed [53].

Particular attention should be paid to the selection of epoch length, and accelerometer wear time, when designing future study protocols. Colley and colleagues [20] compared 15 s and 60s epoch lengths in a sample of 3- to 5-year-old children and found that applying a 15 s epoch resulted in less TPA (− 64.9 min/day) and LPA (− 69.5 min/day), and more sedentary time (+ 77.4 min/day), than when a 60s epoch was applied. While an optimal epoch length for measuring toddlers’ movement behaviours via accelerometry has not yet been determined, future research in toddlers is needed to examine if a 15 s epoch (recommended for the preschooler cohort) is short enough to accurately capture the sporadic movements of young children [18]. Further, with the majority of studies conducted in this population reporting movement behaviour data as min/day (which is helpful when making comparisons to movement behaviour guidelines globally), accelerometer wear time can play a crucial role when activity data is not presented as a function of wear time. For example, the study from this review with the lowest average wear time (i.e., 8.1 h/day [27]) reported TPA levels of toddlers to be 228 min/day, whereas the study with the highest wear time (i.e., 24 h/day [38]) reported a TPA rate of 636.5 min/day. With the increasing focus on 24-h movement behaviours, future research in this population would benefit from a 24-h accelerometer wear time criterion within its measurement protocol, as wear time can largely influence full-day physical activity and sedentary behaviour estimates and interpretations of whether these young children are meeting guidelines. While compliance to this protocol may pose a challenge with this young population, Santos and colleagues [24] reported that 81.6% of their sample of 202 toddlers had at least 3 days of valid 24-h data.

### Limitations

Firstly, only English-language articles were included in this review, thus potentially limiting the representation of infant and toddler samples from non-English speaking countries. Secondly, as accelerometer use among this young cohort is in its infancy, variability in accelerometer models, sampling intervals, and protocols was evident. Further, not all included toddler studies applied activity intensity cut-points validated in the sample population. This reduced comparability among studies and, as such, true estimates of young children’s movement behaviours may not be reflected. Finally, while all studies included in this review were of high quality, the measurement tool adopted for this review was unable to capture reporting and internal validity characteristics associated with accelerometer protocols. While some studies have created unique quality assessment protocols for their review [21], development and validation of a quality assessment tool for physical activity and sedentary behaviour measurement would greatly benefit this field of research.

### Future research efforts and directions

Infants’ and toddlers’ movement behaviours as measured by accelerometry remain understudied; however, research in this area is rapidly growing. With regard to infants’ physical activity, recent advancements in accelerometry protocols are promising [39, 55]; however, more research is still needed to address external motion recognition (e.g., infants being carried), and appropriate epoch length. Further, infant-specific cut-points that can detect non-ambulatory movement would aid in the interpretation of infants’ movement behaviours; in particular, this would aid in the detection of prolonged sedentary bouts while awake. With regard to tummy time, recent work by Hewitt and colleagues [70] has demonstrated the potential use of accelerometers to detect prone position in infants, which shows promise for objectively determining if infants are meeting the 30 min daily recommendation. The authors suggest more research involving the assessment of infants’ physical exertion while prone is needed in order to elucidate the health benefits of tummy time [70].

While research regarding toddlers’ movement behaviours is growing, toddlers are still being included in preschooler analyses in many studies [71, 72]. While this is often a function of how different jurisdictions define the toddler and preschooler age groups (e.g., preschooler classrooms in childcare centres may start at 24 months), physical activity researchers should aim to report age-specific data for more accurate comparison to guidelines. Further, more consistent accelerometer protocols (including wear time, monitoring time, device type and placement, epoch length, cut-points applied, and treatment of naps throughout the day) would aid in interpreting estimates across studies. It would also be beneficial to study 24-h movement behaviours globally to determine if the interaction among sleep, sedentary behaviour, LPA, and MVPA differs by region with regard to proportional estimates, as well as the effects of movement profiles on health markers. Additionally, contextual information regarding the types of sedentary activities toddlers engage in would help with the interpretation of sedentary time estimates, as some sedentary behaviours offer more educational value than others. Moreover, it would be beneficial to explore the childcare environment as a platform for intervention within this population, as childcare centre characteristics have consistently been associated with physical activity rates in preschoolers [73]. Education and promotion of the 24-h movement guidelines among parents, guardians, pediatricians, and early childhood educators may benefit young children in developing and maintaining healthy movement profiles.

## Conclusion

As the first systematic review and meta-analysis to summarize infants’ and toddlers’ accelerometry-measured physical activity and sedentary time, this study contributes greatly to the growing area of movement behaviour research in young children (< 3 years) and is timely with the recent release of 24-h movement guidelines in Canada [11], Australia [12], and New Zealand [13]. Due to the limited studies conducted in infants, physical activity estimates were difficult to ascertain. Further, without validated cut-points for this age group, these results cannot be meaningfully translated into daily rates. In general, toddlers seem to be exceeding their TPA recommendation of 180 min/day; however, the majority of studies reported MVPA estimates below the 60 min/day recommendation for the preschooler cohort, representing an area for improvement. Additionally, a substantial proportion of toddlers’ waking hours were spent in sedentary behaviour. Considering the substantial variability within accelerometer protocols among included studies (e.g., epoch length, device type and placement, and cut-points applied), more consistent and valid protocols for accelerometry-based measurement of toddlers’ movement behaviours should be developed and adopted globally in order to produce more precise estimates that can be compared across studies.

## Supplementary information

**Additional file 1: Table S1**. Sample Search Strategy (EMBASE).

**Additional file 2: Table S2.** Quality Assessment for Included Studies (*n* = 24).

## Data Availability

The dataset generated and analyzed during the present study is available from the corresponding author upon reasonable request.

## Abbreviations

IQR: Interquartile range
LPA: Light physical activity
MVPA: Moderate-to vigorous-intensity physical activity
SD: Standard deviation
TPA: Total physical activity
