# Supplementary material for: Infants’ and toddlers’ physical activity and sedentary time as measured by accelerometry: a systematic review and meta-analysis
Source: Int J Behav Nutr Phys Act. 2020 Feb 7;17:14. doi: 10.1186/s12966-020-0912-4 (PMC7006115; doi:10.1186/s12966-020-0912-4)
Supplement: Supplementary file 1 — Additional file 1: Table S1. Sample Search Strategy (EMBASE). [file 12966_2020_912_MOESM1_ESM.docx]

Supplementary Table 1. *Sample Search Strategy (EMBASE)*

| # | Search Term | Results | Search Type |
| --- | --- | --- | --- |
| 1 | exp toddler/ | 3804 | Advanced |
| 2 | “early years”.mp. | 4690 | Advanced |
| 3 | childhood/ | 82147 | Advanced |
| 4 | infant*.mp. | 937940 | Advanced |
| 5 | preschool child/ | 582165 |  |
| 6 | “young child*”.mp. | 62770 | Advanced |
| 7 | 1 or 2 or 3 or 4 or 5 or 6 | 1377389 | Advanced |
| 8 | exercise/ or “physical activity, capacity and performance”/ or aerobic exercise/ or exercise intensity/ | 287487 | Advanced |
| 9 | “physical activity”.mp. or physical activity/ | 180385 | Advanced |
| 10 | movement.mp. | 392099 | Advanced |
| 11 | active play.mp. or motor activity/ | 48405 | Advanced |
| 12 | locomotion.mp. or locomotion/ | 79769 | Advanced |
| 13 | locomotor activity.mp. | 25660 | Advanced |
| 14 | active movement.mp. | 1400 | Advanced |
| 15 | outdoor play.mp. | 268 | Advanced |
| 16 | outdoor time.mp. | 97 | Advanced |
| 17 | recess.mp. | 5332 | Advanced |
| 18 | sedentary lifestyle/ or sedentary behavio*.mp. | 14818 | Advanced |
| 19 | sedentary.mp. | 40145 | Advanced |
| 20 | inactive.mp. | 112863 | Advanced |
| 21 | physical inactivity.mp. or physical inactivity/ | 10970 | Advanced |
| 22 | sedentary activity.mp. or sitting/ | 21979 | Advanced |
| 23 | 8 or 9 or 10 or 11 or 12 or 13 or 14 or 15 or 16 or 17 or 18 or 19 or 20 or 21 or 22 | 1043869 | Advanced |
| 24 | accelerometer/ or acceleromet*.mp. or accelerometry/ | 19395 | Advanced |
| 25 | actigraph.mp. | 3685 | Advanced |
| 26 | actical.mp. | 323 | Advanced |
| 27 | actiheart.mp. | 181 | Advanced |
| 28 | activpal.mp. | 327 | Advanced |
| 29 | 24 or 25 or 26 or 27 or 28 | 21233 | Advanced |
| 30 | 7 and 23 and 29 | 796 | Advanced |
