# Supplementary material for: Infants’ and toddlers’ physical activity and sedentary time as measured by accelerometry: a systematic review and meta-analysis
Source: Int J Behav Nutr Phys Act. 2020 Feb 7;17:14. doi: 10.1186/s12966-020-0912-4 (PMC7006115; doi:10.1186/s12966-020-0912-4)
Supplement: Supplementary file 2 — Additional file 2: Table S2. Quality Assessment for Included Studies (n = 24). [file 12966_2020_912_MOESM2_ESM.docx]

| Supplementary Table 2. *Quality Assessment for Included Studies (n = 24)* | | | | | | | | | | |  |
| --- | --- | --- | --- | --- | --- | --- | --- | --- | --- | --- | --- |
| **Study** | **Q1** | **Q2** | **Q3** | **Q6** | **Q7** | **Q10** | **Q11** | **Q12** | **Q18** | **Q20** | **Total** |
| Armstrong et al. (2018) | 1 | 1 | 1 | 1 | 1 | 1 | 1 | 1 | 1 | 1 | **10** |
| Bisson et al. (2018) | 1 | 1 | 1 | 1 | 1 | 1 | 1 | 1 | 1 | 1 | **10** |
| Borkhoff et al. (2015) | 1 | 1 | 1 | 1 | 1 | 1 | 1 | 1 | 1 | 1 | **10** |
| Dlugonski et al. (2017) | 1 | 1 | 1 | 1 | 1 | 1 | 1 | 0 | 1 | 1 | **9** |
| Hager et al. (2016) | 1 | 1 | 1 | 1 | 1 | 1 | 1 | 0 | 1 | 1 | **9** |
| Hauck & Felzer-Kim (2019) | 1 | 1 | 1 | 1 | 1 | 1 | 1 | 1 | 1 | 1 | **10** |
| Herzig et al. (2017) | 1 | 1 | 1 | 1 | 1 | 1 | 1 | 0 | 1 | 0 | **8** |
| Hnatiuk et al. (2012) | 1 | 1 | 1 | 1 | 1 | 1 | 1 | 1 | 1 | 1 | **10** |
| Hnatiuk et al. (2017) | 1 | 1 | 1 | 1 | 1 | 0 | 1 | 0 | 1 | 1 | **8** |
| Jia et al. (2018) | 1 | 1 | 1 | 1 | 1 | 1 | 1 | 1 | 1 | 1 | **10** |
| Johansson et al. (2016) | 1 | 1 | 1 | 1 | 1 | 1 | 1 | 1 | 1 | 1 | **10** |
| Konstabel et al. (2014) | 1 | 1 | 1 | 1 | 1 | 0 | 1 | 1 | 1 | 1 | **9** |
| Kwon et al. (2019) | 1 | 1 | 1 | 1 | 1 | 1 | 1 | 1 | 1 | 1 | **10** |
| Lee et al. (2017) | 1 | 1 | 1 | 1 | 1 | 0 | 1 | 1 | 1 | 1 | **9** |
| McCullough et al. (2018) | 1 | 1 | 1 | 1 | 1 | 1 | 1 | 1 | 1 | 1 | **10** |
| Oftedal et al. (2015) | 1 | 1 | 1 | 1 | 1 | 1 | 1 | 0 | 1 | 1 | **9** |
| Pitchford et al. (2017) | 1 | 1 | 1 | 1 | 1 | 1 | 1 | 0 | 1 | 1 | **9** |
| Pulakka et al. (2017) | 1 | 1 | 1 | 1 | 1 | 1 | 1 | 1 | 1 | 1 | **10** |
| Santos et al. (2017) | 1 | 1 | 1 | 1 | 1 | 1 | 1 | 1 | 1 | 1 | **10** |
| Taylor et al. (2018) | 1 | 1 | 1 | 1 | 1 | 0 | 1 | 1 | 1 | 1 | **9** |
| Tsai et al. (2011) | 1 | 1 | 1 | 1 | 1 | 0 | 1 | 1 | 1 | 1 | **9** |
| Vanderloo et al. (2015) | 1 | 1 | 1 | 1 | 1 | 1 | 1 | 1 | 1 | 1 | **10** |
| Wang et al. (2019) | 1 | 1 | 1 | 1 | 1 | 1 | 1 | 1 | 1 | 1 | **10** |
| Wijtzes et al. (2013) | 1 | 1 | 1 | 1 | 1 | 0 | 1 | 1 | 1 | 1 | **9** |
